# Supplementary material for: The interactome of intact mitochondria by cross-linking mass spectrometry provides evidence for coexisting respiratory supercomplexes
Source: Mol Cell Proteomics. 2017 Dec 8;17(2):216–32. doi: 10.1074/mcp.RA117.000470 (PMC5795388; doi:10.1074/mcp.RA117.000470)
Supplement: Supplemental Data [file supp_RA117.000470_133922_0_supp_23978_fzffwf.zip › spectra_annotation/mito_spectra_annotation/251-1-1-1-3-1.pdf]

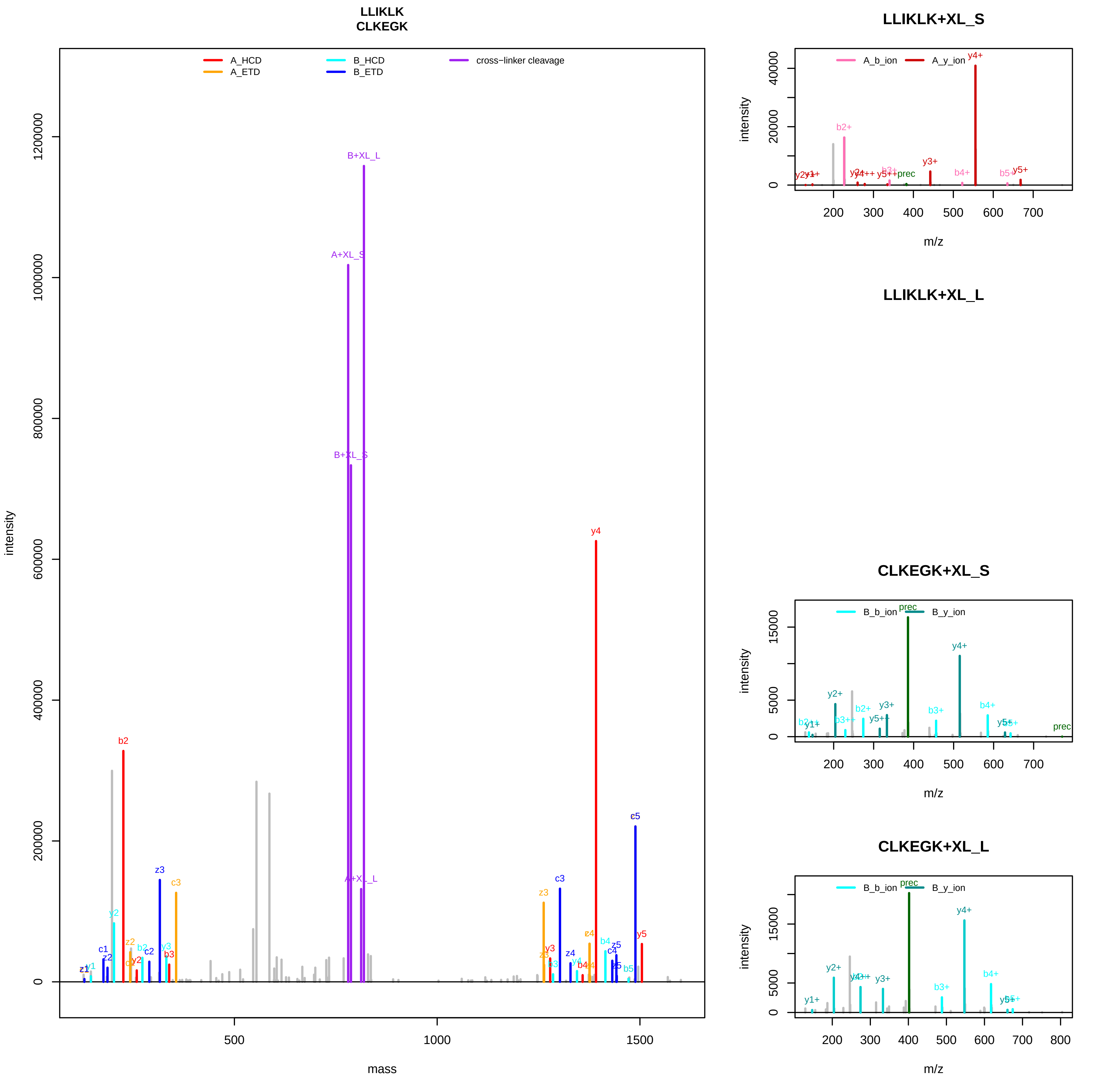

LLIKLK
CLKEGK
A_HCD
A_ETD
B_HCD
B_ETD
cross−linker cleavage
intensity
mass
0
200000
400000
600000
800000
1000000
1200000
500
1000
1500
B+XL_L
A+XL_S
B+XL_S
A+XL_L
y4
c5
y5
b2
y2
z3
c3
c1
z2
c2
b2
y3
b3
z1
y1
z2
y2
c3
z3
y3
c3
z4
y4
b4
z4
b4
c4
z5
b5
LLIKLK+XL_S
A_b_ion
A_y_ion
y4+
b2+
y3+
b4+
b5+
y5+
prec
intensity
m/z
0
20000
40000
200
300
400
500
600
700
LLIKLK+XL_L
CLKEGK+XL_S
B_b_ion
B_y_ion
prec
y4+
y2+
b2+
y3+
b3+
b4+
y1+
intensity
m/z
0
5000
15000
200
300
400
500
600
700
CLKEGK+XL_L
B_b_ion
B_y_ion
prec
y4+
y2+
y3+
b3+
b4+
y1+
intensity
m/z
0
5000
15000
200
300
400
500
600
700
800
